# Advanced QuEChERS Method Using Core-Shell Magnetic Molecularly Imprinted Polymers (Fe_3_O_4_@MIP) for the Determination of Pesticides in Chlorophyll-Rich Samples

**DOI:** 10.3390/foods12203742

**Published:** 2023-10-11

**Authors:** Zhen-Peng Kai, Meng-Xia Hou, Jing-Jing Zhu, Zhong-Ping Jiang, Shan-Shan Chen

**Affiliations:** 1 School of Chemical and Environmental Engineering, Shanghai Institute of Technology, Shanghai 201418, China; kaizp@sit.edu.cn (Z.-P.K.); mengxiahou0406@163.com (M.-X.H.); 15212123252@163.com (J.-J.Z.); 2 Shandong Province Key Laboratory of Chemical Pesticide, Shandong Academy of Pesticide Sciences, Jinan 250100, China; jzp7625879@163.com; 3 Institute of Agro-Food Standards and Testing Technologies, Shanghai Academy of Agricultural Sciences, Shanghai 201403, China

**Keywords:** planar and aromatic pesticide residues, chlorophyll, QuEChERS, Fe_3_O_4_@MIP

## Abstract

Graphitized carbon black (GCB) in the traditional QuEChERS (quick, easy, cheap, effective, rugged, and safe) method was used to remove the interfering substance chlorophyll in vegetable and fruit samples for pesticide residues determination. However, it not only adsorbs pigments, but also adsorbs some planar and aromatic pesticides. In order to solve the shortcoming, a core-shell magnetic molecularly imprinted polymer (Fe_3_O_4_@MIP) that can specifically recognize and adsorb chlorophyll was synthesized, and an advanced QuEChERS method with the Fe_3_O_4_@MIP as a purification material was developed. This advanced method presents detection that is highly sensitive, specific, and reproducible for planar and aromatic pesticides. The limits of detection (LOD) ranged from 0.001–0.002 mg kg^−1^, and the limit of quantification (LOQ) was 0.005 mg kg^−1^. The recovery for the planar and aromatic pesticides was within 70–110% with the associated relative standard deviations < 15% in leek samples by the advanced QuEChERS method. However, in the traditional QuEChERS method with GCB, the recovery of most planar and aromatic pesticides was <60%. It may also be useful for the determination of other pesticides in vegetable samples with quick and easy sample purification.

## 1. Introduction

Pesticides are considered necessary and essential for the cultivation and storage of crops, vegetables, fruits, and other agricultural products. However, pesticide residues that may pose a potential risk to human health are a matter of public concern [1]. In order to meet the public’s requirements, pesticide residue laboratories have to provide information on large amounts of analytes in a short period of time. In pesticide residues detecting, sample preparation is crucial because it may accelerate the speed of the whole measurement. Scientists have developed numerous sample preparation methods to achieve this purpose. The QuEChERS sample preparation approach became very popular in many laboratories due to its advantages in comparison with other techniques [2]. It stands as a pivotal advancement in modern analytical chemistry, revolutionizing the way samples are prepared for analysis. This innovative approach offers a rapid, cost-effective, and robust solution for the extraction and clean-up of complex matrices, making it an indispensable tool in various scientific fields. It has been successfully used to determine multi-pesticide residues in food products, feedstuff, and environmental samples [3].

GCB is often used to remove the interfering substance chlorophyll during the purification procedure of vegetable samples [4]. However, it not only adsorbs pigments such as chlorophyll, but also adsorbs some planar and aromatic pesticides—hexachlorobenzene, tolylfluanid, and thiabendazole [5,6,7]. This affects the accuracy of planar and aromatic pesticide residues detection. Therefore, we need to develop new materials to replace GCB, which only adsorbs chlorophyll and does not adsorb pesticide residues.

Molecularly imprinted polymers (MIPs) are synthetic tailor-made polymers which can selectively recognize or extract target analytes in a complicated matrix [8]. MIPs have been widely used for the isolation, separation, and monitoring of trace substances from different samples, such as vegetables, fruits, food products, and environmental samples with high affinity, selectivity, and stability [9,10]. In pesticide residue analysis, MIPs are often applied for the detection of a specific molecule or a family of compounds [11,12,13,14,15].

In order to solve the shortcomings of GCB that adsorbs some planar and aromatic pesticides, a core-shell magnetic MIP (Fe_3_O_4_@MIP) was developed for selective removal of chlorophyll from samples. The QuEChERS method was modified with this Fe_3_O_4_@MIP material which replace GCB to detect the pesticides, especially the planar and aromatic pesticides, in chlorophyll-rich samples.

## 2. Materials and Methods

### 2.1. Reagents and Materials

FeCl_3_·6H_2_O and FeCl_2_·4H_2_O was purchased from Xilong Chemical Industry Incorporated (Shantou, China). NH_2_·H_2_O was purchased from Tianjin Chemical Works (Tianjin, China). Chlorophyll, hemin, methacrylic acid (MAA), ethylene glycol dimethacrylate (EGDMA), and 2, 2-azobisisobutyronitrile (AIBN) were supplied by Accelerating Scientific and Industrial Development thereby Serving Humanity (Shanghai, China). Anhydrous magnesium sulphate (MgSO_4_), PSA, and GCB were purchased from Agela Technologies Inc. The leek samples were obtained from a supermarket (Shanghai, China).

**Figure 1:**
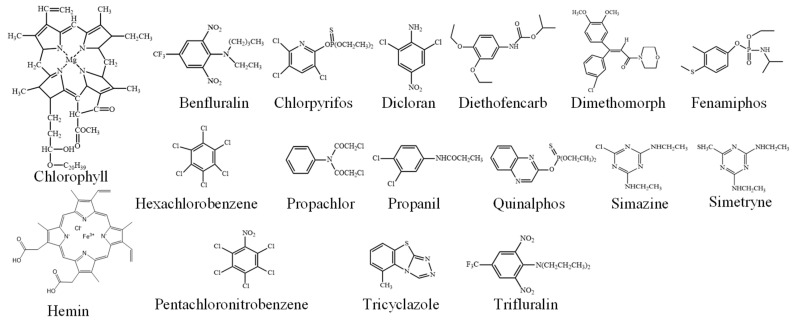
Structures of chlorophyll, hemin, planar, and aromatic pesticides [17].

The standards of the planar and aromatic pesticides (Benfluralin, Chlorpyrifos, Dicloran, Diethofencarb, Dimethomorph, Fenamiphos, Hexachlorobenzene, Pentachloronitrobenzene, Propachlor, Propanil, Quinalphos, Simazine, Simetryne, Tricyclazole, and Trifluralin, structures shown in Figure 1) were purchased from Dr. Ehrenstorfer GmbH (Augsburg, Germany) and Sigma-Aldrich (Saint Louis, MO, USA). Standard stock solutions of pesticides were prepared in acetone or methanol and stored at −20 °C (1000 mg L^−1^). The working multi-standard solutions at the appropriate concentrations were prepared by dilution with acetone or methanol [16].

### 2.2. Preparation of Fe_3_O_4_ Nanoparticles (NPs)

First, 15 mL ammonia and 237 mL ultrapure water were mixed in a dry 500 mL three-necked flask at 35 °C. The mixture was stirred and removed O_2_ from the solution by N_2_. A solution of FeCl_3_·6H_2_O (6.76 g, 25 mmol) and FeCl_2_·4H_2_O (1.99 g, 10 mmol) in 30 mL ultrapure water was added drop by drop. The reaction mixture was stirred at 35 °C under a nitrogen atmosphere for 1 h. Then, the products were separated by super-magnets, washed 5 times with ultrapure water, and dried in a vacuum drying oven at 60 °C for 12 h.

### 2.3. Synthesis of Methacrylic Acid (MAA)-Modified

First, 0.2 g Fe_3_O_4_ and 10 mL toluene were added in a dry round-bottom flask. After ultrasonic dispersion for 30 min, 2 mL methacrylic acid (MAA) was added, and then stirred at 30 °C for under a nitrogen atmosphere 24 h. After reaction completion, the obtained modified Fe_3_O_4_ (Fe_3_O_4_–MAA) was separated by super-magnets, washed with ultrapure water and ethanol, and then dried in a vacuum drying oven at 60 °C for 12 h. 

### 2.4. Preparation of the Fe_3_O_4_@MIPs and Fe_3_O_4_@NIPs

First, 0.2 g Fe_3_O_4_–MAA, 0.0686 g hemin, 0.0334 g MAA, and 30 mL acetonitrile were mixed in a dry round-bottom flask, then ultrasonic dispersed for 30 min. After dispersion, 360 µL of ethylene glycol dimethacrylate (EGDMA) and 40 mg of azobisisobutyronitrile (AIBN) were added to the flask. N_2_ was used for removal of oxygen from the solution. The flask was then sealed with a solid rubber stopper, and polymerization was carried out at 60 °C in shaking bath for 24 h. After reaction completion, the imprinted polymer was separated by super-magnets, washed with ultrapure water and a mixture of methanol/acetic acid (*v*/*v*, 9:1) for removing the template molecule, then dried in a vacuum drying oven at 60 °C for 12 h.

The preparation of magnetic non-molecular imprinted material Fe_3_O_4_@NIP was similar to Fe_3_O_4_@MIP. The only difference was that template hemin was not added before polymerization.

### 2.5. Adsorption Capacity

The adsorption capacity of Fe_3_O_4_@MIP and Fe_3_O_4_@NIP was evaluated using chlorophyll as the test compounds of 5 mg prepared Fe_3_O_4_@MIP and Fe_3_O_4_@NIP were separately added into 15 mL centrifuge tubes, and 10 mL chlorophyll ethanol solution with initial concentration of 30, 60, 120, 180, 240, 300, 360, 420, 480, 540, 600, 660, 720 mg L^−1^ were added. Shaken at room temperature in an air shaker for 2.5 h, the supernatant was separated by super-magnets. The chlorophyll concentration in the supernatant was measured by UV-Vis spectrometer with detection wavelength 670 nm.

The process of dynamic adsorption experiment was similar to the above description. First, 20 mg prepared Fe_3_O_4_@MIP and Fe_3_O_4_@NIP were separately added into 40 mL chlorophyll acetonitrile solution (the concentration was 540 mg L^−1^). These samples were fixed separately on an air shaker and oscillated at room temperature for different times. According to the time points, the supernatants were separated by super-magnets at 5, 10, 20, 30, 40, 50, 60, 80, 100, and 120 min. The chlorophyll concentration in the supernatant was measured as above.

### 2.6. Sample Preparation

The recovery experiments of the target planar and aromatic pesticides were carried out to compare the adsorption ability of Fe_3_O_4_@MIP and GCB. Sample preparations and purifications were carried out by QuEChERS citric acid buffer (EN method) [18,19]. Homogenous leek samples weighing 10 g were placed into 50 mL centrifuge tubes, and the appropriate pesticide mixed standard solutions were added to obtain the expected levels and maintained for 30 min. After 10 mL acetonitrile and the QuEChERS citric acid extraction salt packet (4 g anhydrous MgSO_4_, 1 sodium citrate, 1 g NaCl, and 0.5 g sodium hydrogen citrate) were added, the mixtures were immediately vortexed vigorously for 1.0 min, and centrifuged at 4000 rpm for 10 min.

To compare the differences in the recoveries of planar structured pesticides obtained by purifying chlorophyll with Fe_3_O_4_@MIP or GCB, the 8 mL supernatant was divided into 4 mL as two equal groups, transferred into the 15 mL centrifuge tube containing (1) 900.0 mg anhydrous MgSO_4_, 150.0 mg PSA, and 45.0 mg GCB or (2) 900.0 mg anhydrous MgSO_4_, 150.0 mg PSA, and 45.0 mg Fe_3_O_4_@MIP. They were then vortexed and purified with the sorbents for 1 min. The mixtures were separated under an outer magnetic field for 3 s, and then the solutions for purification treatment by two different materials were centrifuged at 4000 rpm for 10 min, respectively. Finally, 1 mL of the purified sample solutions from two groups was filtered through a 0.22 µm nylon membrane for UPLC-MS/MS analysis, the other 1 mL supernatant was evaporated to dryness under a stream of nitrogen at 40 °C, and the residue was redissolved in 1 mL of acetone for GC-MS/MS analysis.

### 2.7. GC-MS/MS Analysis

GC-MS/MS analysis was performed using a Thermo Scientific Trace 1310 GC, coupled with a TSQ 9000 Triple Quadrupole MS (Thermo Fisher Scientific Technologies, Waltham, MA, USA). A Thermo Scientific’s TG-5 SILMS capillary column (0.25 mm i.d. ×30 m, 0.25 µm film thickness) was used to provide analyte separation. The column was set at a constant flow rate of 1 mL min^−1^ using helium as carrier gas. A volume of 2 µL was injected in splitless mode through an ultrainert inlet liner tube with a glass wool frit. The temperature of the injector was 250 °C, and the ion source and transfer line was set at 280 °C. The column temperature was programmed as follows: the initial temperature was 60 °C, and raised to 90 °C at 30 °C min^−1^, 180 °C at 15 °C min^−1^, then to 250 °C at 7 °C min^−1^, and then to 280 °C at 30 °C min^−1^, held for 2 min; the total running time was 20 min. The two optimal ion transitions for multiple reaction monitoring (MRM) of each pesticide were determined by collision tests, i.e., primary and secondary transitions from precursors to productions [20]. Quantitation by GC-MS/MS was based on the TraceFinder 4.1 software. Identification of pesticides in fortified samples was determined by comparing the expected retention time (*t*_R_) and the ratio of the two transition (primary/secondary) results to matrix-matched standards. The specific MRM transitions for the test pesticides and other parameters were given in Table S1.

### 2.8. UPLC-MS/MS Analysis

The assay was performed on the Acquity UPLC (Waters) system connected to the AB SCIEX 5500 triple-quadrupole mass spectrometer (Framingham, MA, USA). The target analytes were isolated on a BEH C18 column (100 mm × 2.1 mm, 1.7 µm particle size) maintained at 30 °C. The mobile phases were methanol (A) and 0.1% HCOOH in ultrapure water (B), and the flow rate was 0.3 mL min^−1^. The gradient program started at 10%, with a hold for 1 min, then was changed to 90% A (in 1–6 min), and finally decreased to 10% A (6–7 min). The injection volume was 5 µL. The ion spray voltage was set at 4500 V, 99.99% N_2_ was used as the desolvation/nebulizer gas, and 99.99% Ar as the collision gas. The temperature of the block source was maintained at 500 °C, while the pressure of nebulizer gas and turbo gas was set at 45 psi. The curtain gas pressure was 40 psi, and the collision gas value was set to 8. AB Sciex v1.6 analyst software was used for controlling instruments, data acquisition, and processing. The optimal MRM transitions and other parameters of UPLC-MS/MS for the test pesticides were given in Table S2.

## 3. Results and Discussion

### 3.1. Preparation and Characterization of Fe_3_O_4_@MIP

**Figure 2:**
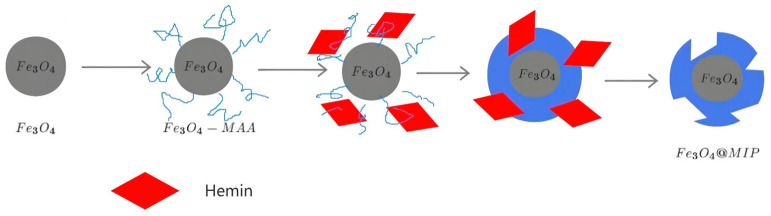
Schematic illustration of the synthesis of Fe_3_O_4_@MIP.

The general scheme for the synthesis of magnetic molecular imprinting material is shown in Figure 2. First, Fe_3_O_4_ nanoparticles were synthesized by co-precipitation. Then, methacrylic acid was grafted onto the nanoparticles to achieve surface modification of the nanoparticles. The modified nanoparticles were blended with methacrylic acid monomer, template molecule hemin, initiator, and crosslinking agent to initiate polymerization to form the polymer. The template molecules in the polymer were eluted to obtain a magnetic molecular imprinting material. Due to the easy degradation of chlorophyll, we chose a similar structure of hemin as a template molecule. The molecular imprinting material can selectively adsorb chlorophyll in the solvent.

**Figure 3:**
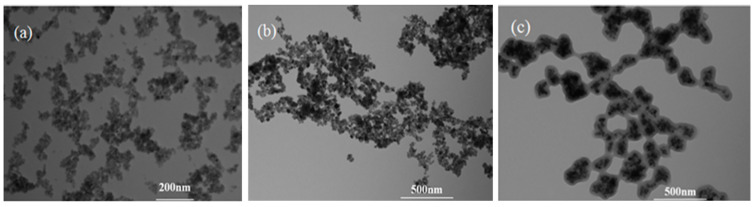
SEM images of (**a**) Fe_3_O_4_ nanoparticles; (**b**) MAA–Fe_3_O_4_; (**c**) Fe_3_O_4_@MIP.

The morphology of the Fe_3_O_4_@MIP was observed with scanning electron microscopy (SEM). The iron oxide nanoparticles, the nanoparticles modified by MAA, and the magnetic molecular imprinting materials are shown in Figure 3. Fe_3_O_4_ nanoparticles were approximately spherical, with a diameter of about tens of nanometers. After the surface modification, the thickness of the ball had increased significantly, because the surface of the nanoparticles was wrapped in a layer of methacrylic acid. Nanoparticles do not have very serious agglomeration. In particular, after polymerization, it was evident that the polymer encapsulates the nanoparticles to form a core-shell composite (shown in Figure 3c).

**Figure 4:**
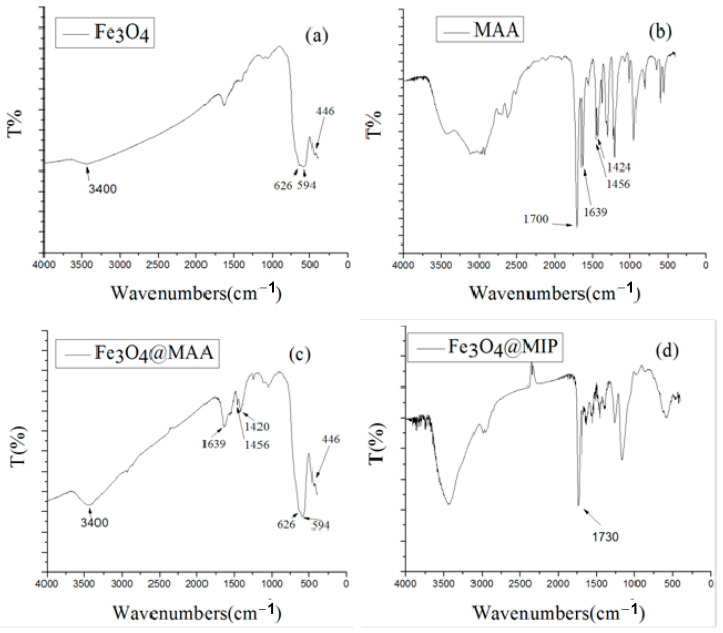
FT-IR spectra of (**a**) Fe_3_O_4_; (**b**) MAA; (**c**) Fe_3_O_4_@MAA; (**d**) Fe_3_O_4_@MIP.

Fourier-transform infrared (FT-IR) spectroscopy was performed to further characterize the Fe_3_O_4_ nanoparticles, MAA, and Fe_3_O_4_@MIP (Figure 4). For the Fe_3_O_4_ nanoparticles, the strong absorption peaks at 626, 594, and 446 cm^−1^ are typical Fe–O absorption bands in Fe_3_O_4_. For the MAA, the strong band at 1700 cm^−1^ was the stretching vibrations of C=O in carboxylic acid dimer, the adsorption peaks at 1639 cm^−1^ were assigned to the stretching vibration of C=C, and the adsorption peaks at 1456 mL^−1^ and 1424 cm^−1^ could be attributed to C–H bonds. The characteristic peaks of Fe_3_O_4_@MIP are shown in Figure 4d. The strong peak around 1730 cm^−1^ of C=O indicated that the cross-linker had been successfully incorporated into the polymers. In addition, the characteristic C=C peaks of 1639 cm^−1^ in MAA disappeared from the Fe_3_O_4_@MIP, indicating that the monomer and MAA were polymerized together to form a polymer. All these results indicated that the Fe_3_O_4_@MIP was successfully grafted on the surface of the Fe_3_O_4_ nanoparticles.

**Figure 5:**
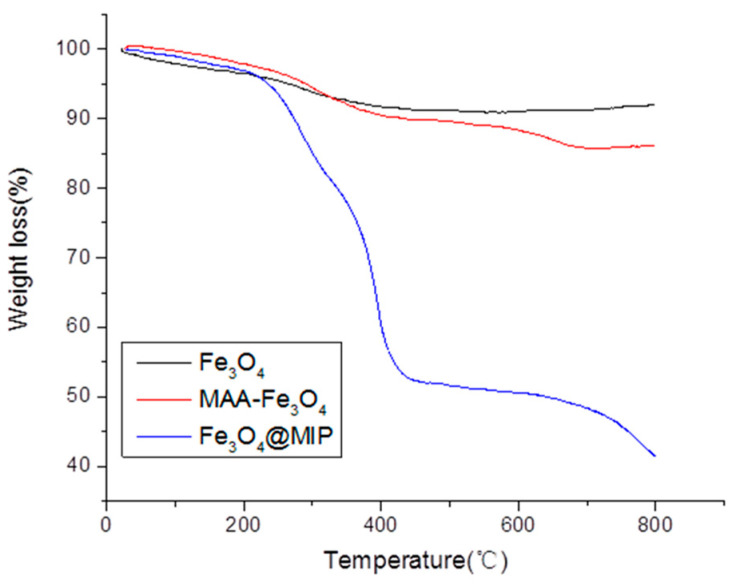
Thermogravimetric analysis of Fe_3_O_4_, MAA−Fe_3_O_4_, and Fe_3_O_4_@MIP.

The thermal stability of Fe_3_O_4_ nanoparticles and Fe_3_O_4_@MIP was performed by thermogravimetric analysis (TGA). As shown in Figure 5, the weight loss of Fe_3_O_4_ nanoparticles was approximately 8% in the temperature range of 30–450 °C due to the loss of residual water in the sample. TGA thermogram of Fe_3_O_4_@MIP showed it was relatively stable (only approximately 5% weight loss) before 250 °C. The significant weight loss of approximately 50% was observed within the range of 250–450 °C for Fe_3_O_4_@MIP. It suggested that the proportion of organic and inorganic materials in Fe_3_O_4_@MIP was close to 1:1. The TGA thermogram also shows that the synthesized Fe_3_O_4_@MIP would be a desirable material in pesticide residue detection.

**Figure 6:**
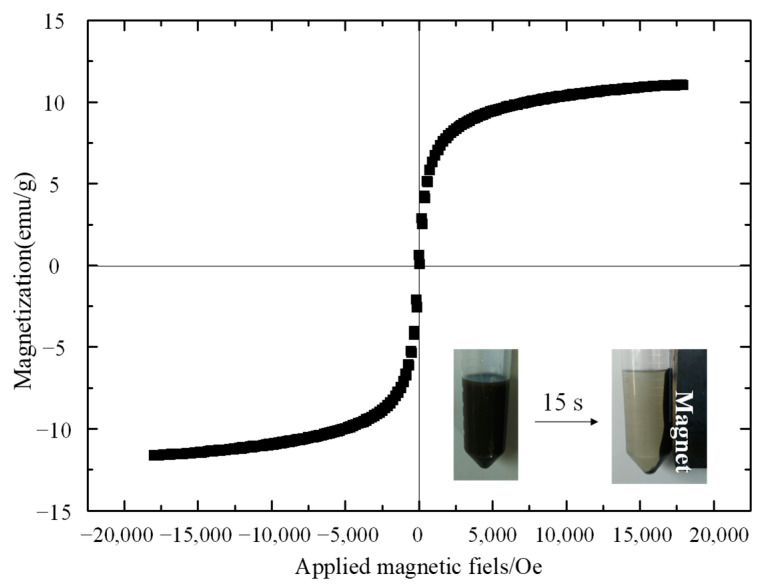
The magnetization curve of Fe_3_O_4_@MIP. The insets show the dispersion and agglomeration processes of Fe_3_O_4_@MIP.

The magnetic separation capability of Fe_3_O_4_@MIP was confirmed by hysteresis loops with VSM and the dispersion/agglomeration process, as shown in Figure 6. The saturation magnetization value of Fe_3_O_4_@MIP was 11 emu g^−1^. When applied as an external magnetic field on the outer side wall of the vials containing chlorophyll solution and Fe_3_O_4_@MIP, the homogeneously dispersed chlorophyll absorbing Fe_3_O_4_@MIP could quickly adhere to the wall of the vial, forming a transparent solution.

### 3.2. Adsorption Capacity of Chlorophyll

**Figure 7:**
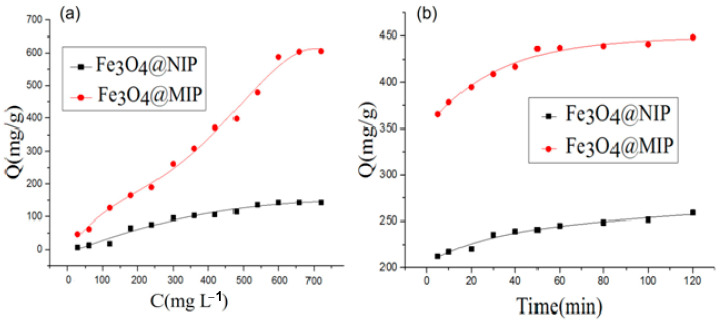
(**a**) Characteristic adsorption isotherms of Fe_3_O_4_@MIP and Fe_3_O_4_@NIP; (**b**) Adsorption kinetics of Fe_3_O_4_@MIP and Fe_3_O_4_@NIP.

The static adsorption capacity of the Fe_3_O_4_@MIP and Fe_3_O_4_@NIP were investigated with a range of standard chlorophyll solutions, ranging from 30 to 720 mg L^−1^. In Figure 7a, the adsorptive maximum capacity of Fe_3_O_4_@MIP (up to 628.9 mg L^−1^) was 5.0 times that of Fe_3_O_4_@NIP (up to 126.3 mg mL^−1^). This indicates that Fe_3_O_4_@MIP exhibits a higher binding affinity for chlorophyll than Fe_3_O_4_@MIP, which shows the specific recognition sites were successfully established during the molecular imprinting process. Figure 7b shows the adsorption kinetics of Fe_3_O_4_@MIP and Fe_3_O_4_@NIP. With an increase in extraction time, the adsorption capacities of chlorophyll on Fe_3_O_4_@MIP increased until finally reaching adsorption equilibrium at 60 min. However, Fe_3_O_4_@NIP will take 120 min to reach the adsorption equilibrium. From the adsorption capacity experiments, we can see Fe_3_O_4_@MIP has a stronger adsorption capacity and a faster adsorption rate for chlorophyll.

### 3.3. Purification Effect of the QuEChERS Method Modified with Fe_3_O_4_@MIP

**Figure 8:**
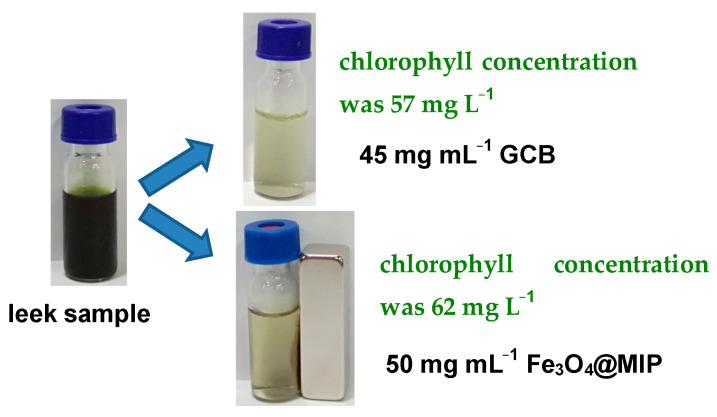
Purification effect of Fe_3_O_4_@MIP and GCB on leek samples.

The advanced QuEChERS method was compared with the traditional QuEChERS for the purification of chlorophyll in vegetable samples. As shown in Figure 8, when the amount of GCB added increased, the chlorophyll content in the leek sample extract gradually decreased. The extract of leek sample prepared in Section 2.6 (the chlorophyll concentration was 6000 mg L^−1^ before adsorption) was used to evaluate the adsorption capacity of GCB and Fe_3_O_4_@MIP, respectively. At 45 mg mL^−1^ of GCB, the chlorophyll in the sample was almost eliminated, and the chlorophyll concentration after adsorption was 57 mg L^−1^. When Fe_3_O_4_@MIP was used as an adsorbent to treat leek samples, the chlorophyll in the sample was substantially removed by 50 mg mL^−1^. After adsorption by Fe_3_O_4_@MIP, the chlorophyll concentration in the sample solution was 62 mg L^−1^. This illustrates that Fe_3_O_4_@MIP has a similar clean-up efficiency compared with GCB during the real vegetable sample processing. When using GCB for purification, the sample needs to be centrifuged and then the supernatant can be removed. However, centrifugation is not necessary when the purification is performed by Fe_3_O_4_@MIP. It reduced the workload of the experimenter during sample preparation for pesticide residues detection.

### 3.4. Linearity, Limit of Detection (LOD), and Limit of Quantification (LOQ)

Ideal linear calibration curves and correlation coefficients (r > 0.99) were observed over the concentration range from 0.001–0.4 mg L^−1^. The matrix-matched calibration standard solutions were prepared in order to avoid matrix effect errors in analysis. All experiments were performed with five replicates to determine the concentration by comparing the peak area in the sample with the peak area of a matrix-matched standard prepared at a known concentration. In this study, we defined LOD as the minimum concentration levels at which the matrix matched the standard curve and set LOQ to the lowest verified concentration that could be quantified with acceptable accuracy and precision. As shown in Table 1, the results were satisfactory according to the guidelines for the national standards of pesticide residue detection methods [21].

### 3.5. Effect of Fe_3_O_4_@MIP and GCB as Adsorbents on Planar and Aromatic Pesticide Recovery

For recovery studies, prior to extraction and purification, leek samples are added to the corresponding volume of standard solution and allowed to stand for 30 min at room temperature. Five replicate spikes each at 0.005, 0.02, and 0.1 mg kg^−1^ were prepared and processed. In order to compare our advanced method with Fe_3_O_4_@MIP and the traditional QuEChERS method, 45 mg mL^−1^ Fe_3_O_4_@MIP and 50 mg mL^−1^ GCB were selected as adsorbents in the recovery study. As seen in Table 2, the recovery of the test compounds was within 70–120% and the relative standard deviations were <15% in all test matrices with Fe_3_O_4_@MIP. However, in the traditional QuEChERS method with GCB, the recovery of most planar and aromatic pesticides was <60%. For example, the recovery of the planar pesticide hexachlorobenzene at 0.005, 0.02, 0.1 mg kg^−1^ was 90–110% with Fe_3_O_4_@MIP, and <25% with GCB. 

**Figure 9:**
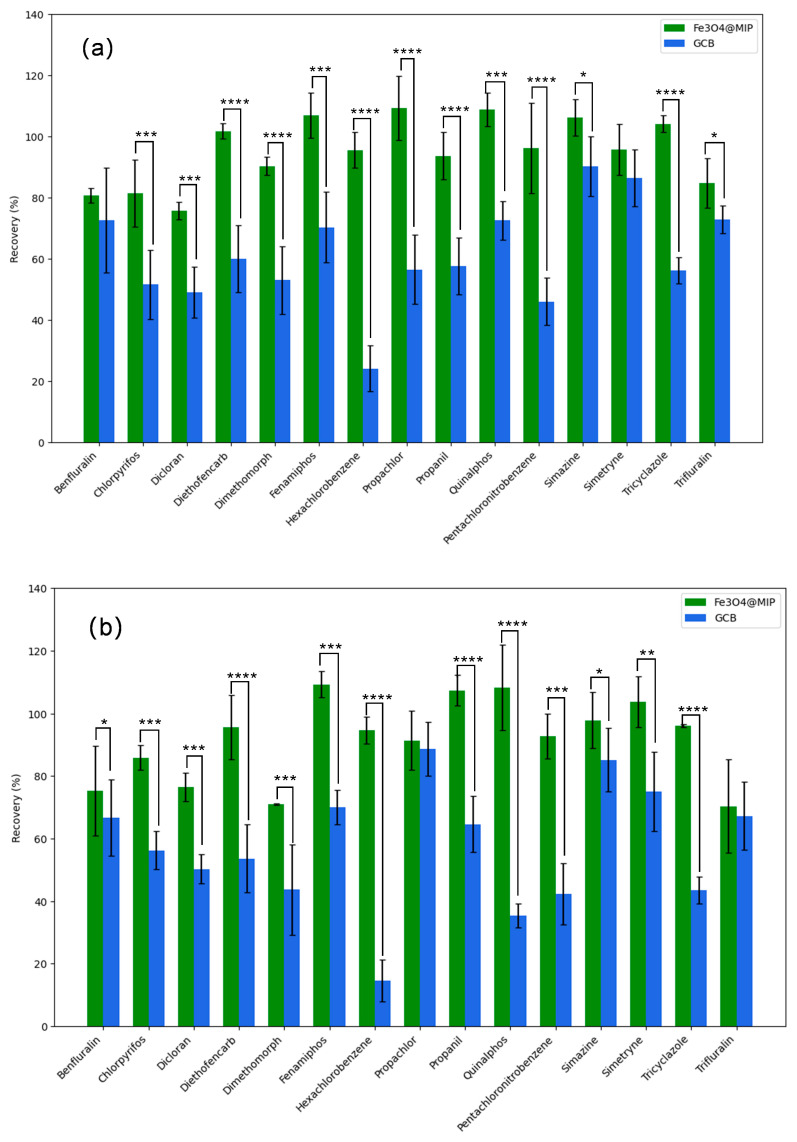

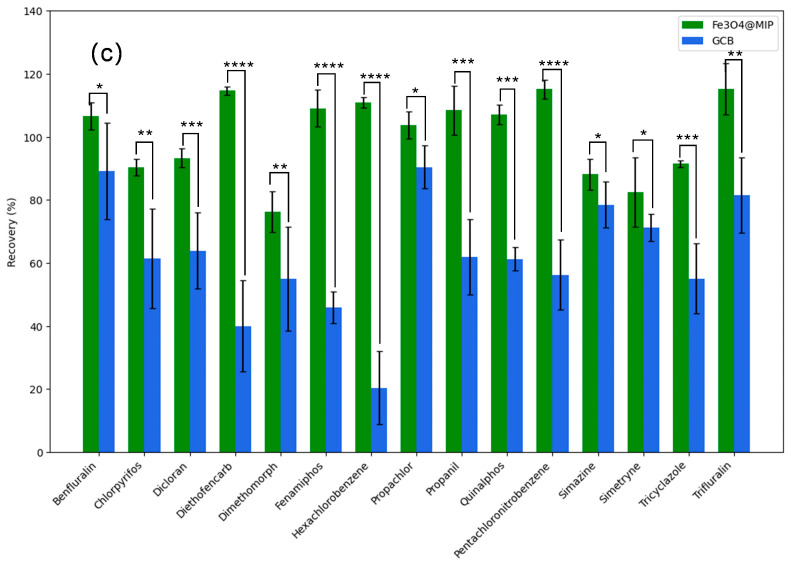
Recovery of Fe_3_O_4_@MIP and GCB as adsorbents at planar and aromatic pesticide with different concentrations of (**a**) 0.005 mg kg^−1^; (**b**) 0.02 mg kg^−1^; (**c**) 0.1 mg kg^−1^. Values represent means ± SD, * *p* < 0.05; ** 0.001< *p* < 0.005; *** 0.0001 < *p* < 0.0005; **** *p* < 0.0001.

In Figure 9, the difference in spiked recoveries at the three concentration levels was significant, with Fe_3_O_4_@MIP being significantly higher than GCB. Statistical analyses were performed with GraphPad Prism version 5.0. A value of 0.05 was used as the threshold for significance. Comparisons of Fe_3_O_4_@MIP and GCB were analyzed with a pooled t-test. When Fe_3_O_4_@MIP was used, the recovery efficiency of some pesticides was effectively increased by several times. For instance, the recovery of dimethomorph, fenamiphos, pentachloronitrobenzene, and hexachlorobenzene obviously improved when using Fe_3_O_4_@MIP other than GCB. This reveals that our material has better selectivity. It only adsorbs the interference chlorophyll but has no effect on the planar and aromatic pesticides. In order to verify whether this method can be widely used for pesticide residue detection, another 117 pesticides were used to conduct the recovery study following our published analysis method. The recovery for the pesticides at 0.005, 0.02, 0.1 mg kg^−1^ ranged from 70 to 120%, and the associated relative standard deviations < 20% in all test matrices (Table S3).

Vegetables and fruits not only contain chlorophyll, but also contain other plant pigments such as lutein, anthocyanins, and carotenes. We can use the same molecular imprinting method to remove these pigments in the detection of multi-pesticide residues. When undertaking the sample preparation of vegetables and fruits rich in multiple pigments, mixing molecular imprinted materials for different pigments will improve the accuracy of detection, especially the detection accuracy of planar and aromatic pesticides. The use of magnetic molecular imprinting materials does not affect the residue detection of non-planar and non-aromatic pesticides, and can reduce the workload of the laboratory analyst.

### 3.6. Conclusions

A core-shell magnetic MIP that can specifically recognize and adsorb chlorophyll was synthesized in this study. The Fe_3_O_4_@MIP exhibited a high adsorption capacity and was successfully employed as a purification material in the advanced QuEChERS method in pesticide residue detection. Compared with the traditional QuEChERS (GCB as the chlorophyll-removing material) method, the advanced method presents high sensitivity, specificity, and is reproducible for planar and aromatic pesticides detection. It can also be widely used for the determination of other pesticides in vegetable and fruit samples.

## Figures and Tables

**Table 1:** Linear Relations, LODs, and LOQs for the Determination of the 15 Planar and Aromatic Pesticides.

Analytes	r^2^	Linear Range (mg L^−1^)	LOD ^a^ (mg kg^−1^)	LOQ ^b^ (mg kg^−1^)
Benfluralin	0.9906	0.001–0.4	0.001	0.005
Chlorpyrifos	0.9998	0.001–0.4	0.001	0.005
Dicloran	0.9962	0.001–0.4	0.001	0.005
Diethofencarb	0.9972	0.001–0.4	0.001	0.005
Dimethomorph	0.9964	0.002–0.4	0.002	0.005
Fenamiphos	0.9924	0.001–0.4	0.001	0.005
Hexachlorobenzene	0.9907	0.001–0.4	0.001	0.005
Pentachloronitrobenzene	0.9946	0.001–0.4	0.001	0.005
Propachlor	0.9928	0.001–0.4	0.001	0.005
Propanil	0.9946	0.002–0.4	0.002	0.005
Quinalphos	0.9910	0.002–0.4	0.002	0.005
Simazine	0.9971	0.001–0.4	0.001	0.005
Simetryne	0.9927	0.001–0.4	0.001	0.005
Tricyclazole	0.9998	0.001–0.4	0.001	0.005
Trifluralin	0.9921	0.002–0.4	0.002	0.005

^a^ Limit of detection; ^b^ Limit of quantification.

**Table 2:** Recoveries (%) and RSDs (%) Obtained from the Analysis of Leek Samples Spiked with Planar and Aromatic Pesticides using Fe_3_O_4_@MIP and GCB.

Analytes	0.005 mg kg^−1^				0.02 mg kg^−1^				0.1 mg kg^−1^			
	Fe_3_O_4_@MIP		GCB		Fe_3_O_4_@MIP		GCB		Fe_3_O_4_@MIP		GCB	
	Recovery(%)	RSD(%)	Recovery (%)	RSD (%)	Recovery (%)	RSD(%)	Recovery (%)	RSD (%)	Recovery (%)	RSD (%)	Recovery (%)	RSD (%)
Benfluralin	80.73	2.4	72.69	17.2	75.21	14.3	66.6	9.2	106.53	4.3	89.03	15.3
Chlorpyrifos	81.46	10.9	51.64	11.3	85.9	4	56.26	6.1	90.28	2.7	61.44	15.7
Dicloran	75.79	2.9	49.01	8.3	76.43	4.7	50.27	4.7	93.26	3	63.88	12
Diethofencarb	101.81	2.6	60.07	11	95.66	10.3	53.63	10.8	114.6	1.3	40.01	14.5
Dimethomorph	90.29	3	52.98	21	71.01	0.2	43.65	33.3	76.27	26.4	54.89	30.1
Fenamiphos	107.03	7.4	70.31	11.5	109.29	4.2	69.99	5.5	109.04	5.8	45.96	5
Hexachlorobenzene	95.59	5.8	24.08	7.5	94.61	4.3	14.51	6.7	110.83	1.7	20.36	11.6
Pentachloronitrobenzene	96.18	14.8	46.01	7.8	92.63	7.2	42.18	9.8	115.07	3.1	56.27	11
Propachlor	109.27	10.5	56.53	21.2	91.38	9.5	88.65	8.5	103.63	4.3	90.35	6.8
Propanil	93.68	7.8	57.65	9.2	107.35	4.9	64.6	9	108.39	7.7	61.91	11.9
Quinalphos	108.92	5.5	72.61	6.3	108.21	13.6	35.4	3.8	107.07	3.2	61.28	3.6
Simazine	106.19	6	90.22	9.8	97.8	9	85.13	10.1	88.06	5	78.42	7.3
Simetryne	95.75	8.4	86.47	9.3	103.67	8.1	75.01	12.6	82.35	11	71.2	4.3
Tricyclazole	104.19	2.8	56.19	4.3	96.05	0.4	43.5	4.3	91.44	1.1	55.01	11.1
Trifluralin	84.9	8	72.84	4.5	70.37	15	67.22	10.8	115.16	8.2	91.43	11.9

## Data Availability

Data is contained within the article or Supplementary Materials.

## Supplementary Materials

The following supporting information can be downloaded at: https://www.mdpi.com/article/10.3390/foods12203742/s1, Table S1: Multiple reaction monitoring (MRM) data acquisition parameters of GC-MS/MS for seven planar and aromatic pesticides; Table S2: Multiple reaction monitoring (MRM) data acquisition parameters of UPLC-MS/MS for the eight planar and aromatic pesticides; Table S3: Recoveries and LOQs for leek at 0.005, 0.02, and 0.1 mg kg^−1^.

## References

[1] Rahman M., Hoque M.S., Bhowmik S., Ferdousi S., Kabiraz M.P., van Brakel M.L. (2021). Monitoring of pesticide residues from fish feed, fish and vegetables in Bangladesh by GC-MS using the QuEChERS method. Heliyon.

[2] Khezri A., Ansari M., Amirahmadi M., Shahidi M., Mohamadi N., Kazemipour M. (2022). Pesticide residues in dates using a modified QuEChERS method and GC-MS/MS. Food Addit. Contam. B.

[3] Anastassiades M., Lehotay S.J., Štajnbaher D., Schenck F.J. (2003). Fast and easy multiresidue method employing acetonitrile extraction/partitioning and “dispersive solid-phase extraction” for the determination of pesticide residues in produce. J. AOAC Int..

[4] Li Y., An Q., Zhang C., Pan C., Zhang Z. (2020). Comparison of Sin-QuEChERS nano and d-SPE methods for pesticide multi-residues in lettuce and Chinese chives. Molecules.

[5] Mastovska K., Dorweiler K.J., Lehotay S.J., Wegscheid J.S., Szpylka K.A. (2010). Pesticide multiresidue analysis in cereal grains using modified QuEChERS method combined with automated direct sample introduction GC-TOFMS and UPLC-MS/MS techniques. J. Agric. Food Chem..

[6] Zhang K., Wong J.W., Hayward D.G., Sheladia P., Krynitsky A.J., Schenck F.J., Webster M.G., Ammann J.A., Ebeler S.E. (2009). Multiresidue pesticide analysis of wines by dispersive solid-phase extraction and ultrahigh-performance liquid chromatography- tandem mass spectrometry. J. Agric. Food Chem..

[7] Lu D., Qiu X., Feng C., Lin Y., Xiong L., Wen Y., Wang D., Wang G. (2012). Simultaneous determination of 45 pesticides in fruit and vegetable using an improved QuEChERS method and on-line gel permeation chromatography–gas chromatography/mass spectrometer. J. Chromatogr. B.

[8] Farooq S., Wu H., Nie J., Ahmad S., Muhammad I., Zeeshan M., Khan R., Asim M. (2022). Application, advancement and green aspects of magnetic molecularly imprinted polymers in pesticide residue detection. Sci. Total Environ..

[9] Chen L., Xu S., Li J. (2011). Recent advances in molecular imprinting technology: Current status, challenges and highlighted applications. Chem. Soc. Rev..

[10] Chen L., Wang X., Lu W., Wu X., Li J. (2016). Molecular imprinting: Perspectives and applications. Chem. Soc. Rev..

[11] Wang A., Lu H., Xu S. (2016). Preparation of magnetic hollow molecularly imprinted polymers for detection of triazines in food samples. J. Agric. Food Chem..

[12] Boulanouar S., Mezzache S., Combès A., Pichon V. (2018). Molecularly imprinted polymers for the determination of organophosphorus pesticides in complex samples. Talanta.

[13] Zhang C., Cui H., Cai J., Duan Y., Liu Y. (2015). Development of fluorescence sensing material based on CdSe/ZnS quantum dots and molecularly imprinted polymer for the detection of carbaryl in rice and Chinese cabbage. J. Agric. Food Chem..

[14] Miao S.S., Wu M.S., Zuo H.G., Jiang C., Jin S.F., Lu Y.C., Yang H. (2015). Core–shell magnetic molecularly imprinted polymers as sorbent for sulfonylurea herbicide residues. J. Agric. Food Chem..

[15] Khan S., Hussain S., Wong A., Foguel M.V., Goncalves L.M., Gurgo M.I.P., Sotomayor M.d.P.T. (2018). Synthesis and characterization of magnetic-molecularly imprinted polymers for the HPLC-UV analysis of ametryn. React. Funct. Polym..

[16] Shao W.C., Zang Y.Y., Ma H.Y., Ling Y., Kai Z.P. (2021). Concentrations and related health risk assessment of pesticides, phthalates, and heavy metals in strawberries from Shanghai, China. J. Food Protect..

[17] Turner J.A. (2021). Pesticide Manual.

[18] He Z., Zhao L., Liu X., Xu Y. (2020). The application of in-source fragmentation in ultra-high performance liquid chromatography-electrospray ionization-tandem mass spectrometry for pesticide residue analysis. J. Chromatogr. A.

[19] He Z., Chen S., Wang L., Peng Y., Luo M., Wang W., Liu X. (2015). Multiresidue analysis of 213 pesticides in leek and garlic using QuEChERS-based method and gas chromatography-triple quadrupole mass spectrometry. Anal. Bioanal. Chem..

[20] Łozowicka B., Rutkowska E., Jankowska M. (2017). Influence of QuEChERS modifications on recovery and matrix effect during the multi-residue pesticide analysis in soil by GC/MS/MS and GC/ECD/NPD. Environ. Sci. Pollut. Res..

[21] (2016). China Pesticide Information Network, Announcement No. 2386 of the Ministry of Agriculture, the People’s Republic of China. http://www.chinapesticide.org.cn/ssfxpg/9556.jhtml.

