# Supplementary material for: Advanced QuEChERS Method Using Core-Shell Magnetic Molecularly Imprinted Polymers (Fe3O4@MIP) for the Determination of Pesticides in Chlorophyll-Rich Samples
Source: Foods. 2023 Oct 11;12(20):3742. doi: 10.3390/foods12203742 (PMC10606496; doi:10.3390/foods12203742)
Supplement: Supplementary file 1 [file foods-12-03742-s001.zip › foods-2624896-supplementary.pdf]

Advanced QuEChERS Method Using Core-shell Magnetic Molecularly Imprinted Polymers (Fe3O4@MIP) for the Determination of Pesticides in Chlorophyll-rich Samples

Table S1. Multiple reaction monitoring (MRM) data acquisition parameters of GC-MS/MS for seven planar and aromatic pesticides.

Pesticides	RTa (min)	Quantification		Confirmation	
		Transition	CEb(ev)	Transition	CEb(ev)
Propachlor	8.95	120.1>77.1	20	176.1>93.0	10
Trifluralin	9.5	264.0>206.2	5	264.0>160.1	15
Benfluralin	9.61	292.1>264.1	10	292.1>206.1	10
Dicloran	9.72	206.1>176.1	10	176.1>148.1	15
Hexachlorobenzene	10.17	283.8>213.9	30	283.8>248.8	15
Pentachloronitrobenzene	10.84	295.0>237.0	20	236.9>142.9	30
Chlorpyrifos	13.27	196.8>168.9	15	198.9>171.0	15

[a] Retention time; [b] Collision energy.

Table S2. Multiple reaction monitoring (MRM) data acquisition parameters of UPLC-MS/MS for the eight planar and aromatic pesticides.

Pesticides	RTa (min)	Quantification			Confirmation		
		Transition	DPb(v)	CEc(v)	Transition	DPb(v)	CEc(v)
Diethofencarb	5.41	268.1>226.1	66	14	268.1>180.1	76	25
Dimethomorph	5.51/5.78	388.1>301.1	120	29	388.1>165.1	105	43
Fenamiphos	5.75	304.1>202	90	45	304.1>217.1	140	31
Propanil	5.55	218.0>162.1	71	21	218.0>127.1	92	37
Quinalphos	5.89	299.1>163.0	50	31	299.1>147.1	77	29
Simazine	4.82	202.1>132.1	55	20	202.1>124.2	55	24
Simetryne	4.5	214.2>124.2	65	28	214.2>96.1	72	36
Tricyclazole	4.34	190.0>163.1	70	32	190.0>136.0	82	38

[a] Retention time; [b] Declustering potential; [c] Collision energy.

Table S3. Recoveries and LOQs for leek at 0.005, 0.02, and 0.1 mg kg^{-1}.

Pesticide	LOQ (μg kg^{-1})	0.005 mg kg^{-1}		0.02 mg kg^{-1}		0.1 mg kg^{-1}	
		Recovery (%)	RSD (%)	Recovery (%)	RSD (%)	Recovery (%)	RSD (%)
Abamectin	10	97.0	2.4	89.6	6.7	88.4	5.5
Acephate	2	80.8	4.6	81.3	6.6	84.2	8.3
Acetamiprid	2	90.3	5.4	107.1	7.8	94.7	6.3
Acetochlor	5	108.7	7.5	91.7	7.3	94.6	7.1
Acrinathrin	2	91.6	5.2	95.5	6.3	96.8	4.9
Alachlor	2	98.5	2.7	92.2	1.8	93.5	4.7
Aldicarb	2	89.6	6.0	106.9	8.4	95.0	4.7

Aldicarb-sulfone	2	88.0	3.7	101.6	3.7	83.0	5.8
Aldicarb-sulfoxide	2	86.1	1.7	94.4	5.0	89.7	4.9
Alpha-HCH	2	101.3	3.1	98.8	1.4	88.3	3.4
Atrazine	2	102.4	4.7	99.5	4.2	90.2	6.2
Azoxystrobin	10	87.3	6.1	99.1	5.5	90.7	3.6
Beta-HCH	2	96.6	8.2	100.7	2.5	94.6	2.7
Bifenthrin	2	94.5	11.8	90.6	3.9	92.3	6.1
Boscalid	2	90.8	2.5	94.8	3.4	92.6	6.7
Bromopropylate	2	91.7	1.9	93.1	2.8	95.4	6.5
Buprofezin	5	93.3	2.9	87.6	10.6	91.3	3.7
Carbaryl	5	95.6	3.2	96.7	6.9	95.9	3.1
Carbendazim	5	94.0	4.0	93.8	6.9	87.0	5.9
Carbofuran	5	94.7	8.2	90.0	9.1	83.7	6.5
Carbophenothion	2	79.3	6.7	100.3	1.8	88.6	6.8
Carboxin	5	88.5	3.9	107.9	4.7	87.7	5.6
Chlorantraniliprole	5	93.6	1.5	98.5	7.5	91.5	3.4
Chlorbenzuron	5	91.1	4.0	104.6	14.2	85.2	6.4
Chlorfenson	2	95.4	2.6	100.3	1.7	91.7	4.6
Chlorfluazuron	5	98.6	3.2	95.9	6.5	85.9	6.1
Chlormequat	2	96.9	10.9	106.0	8.1	94.4	8.0
Chlorpyrifos-methyl	2	92.8	3.5	91.5	4.7	93.6	5.4
Chlorthiophos	2	94.5	3.7	101.6	2.0	92.8	3.6
Clofentezine	5	98.9	3.8	101.5	12.4	84.6	8.3
Clothianidin	5	93.0	2.6	98.9	11.4	88.4	2.5
Cyflufenamid	5	101.6	9.8	95.4	4.3	91.4	8.2
Cyfluthrin	2	92.7	10.9	108.1	3.6	98.7	6.6
Cyhalothrin(lambda)	2	95.3	9.4	110.2	9.2	94.9	7.3
Cypermethrin	2	103.8	3.7	99.1	4.1	95.8	6.5
Cyproconazole	2	95.6	1.4	101.9	1.4	98.6	6.1
Cyprodinil	2	92.9	3.4	101.5	1.9	100.2	4.7
DEF(Tribufos)	2	90.6	8.5	84.2	9.8	91.5	3.6
Delta-HCH	2	99.8	3.1	99.3	6.1	93.7	5.0
Deltamethrin	2	83.9	8.4	93.9	8.5	103.7	5.3
Diazinon	2	95.3	3.6	99.4	3.7	99.4	4.6
Dichlorvos	2	87.1	6.8	80.5	4.3	82.4	5.7
Dicofol	2	91.2	6.4	89.1	3.9	91.7	2.9
Difenoconazole	2	89.8	3.7	105.0	1.4	100.7	4.5
Diflubenzuron	5	90.6	6.5	108.5	8.9	98.3	6.6
Dimethoate	2	85.4	3.2	92.2	1.8	97.6	6.8
Emamectin benzoate	5	98.2	3.4	93.2	5.8	92.4	5.9
Endosulfan	5	90.5	3.6	97.9	8.7	94.3	4.6
Fenhexamid	5	81.8	3.5	84.6	8.3	91.8	6.0
Fenitrothion	2	86.9	5.6	107.5	3.3	106.2	7.5
Fenothiocarb	2	96.2	2.1	93.8	2.9	94.6	6.9

Fenpropathrin	2	92.3	3.4	103.1	2.3	103.5	6.8
Fenthion	2	85.9	1.6	93.4	3.9	91.7	5.6
Fenvalerate	2	88.2	6.9	91.4	7.2	96.7	7.2
Fipronil	2	91.5	8.1	104.8	4.5	93.3	4.7
Fluazinam	5	106.5	5.9	103.3	12.1	92.2	8.3
Flucythrinate	2	80.5	2.9	107.2	2.4	102.1	7.5
Flusilazole	5	90.5	3.6	97.9	8.7	94.3	4.6
Fosthiazate	2	85.3	3.2	99.9	2.5	98.5	5.1
Gamma-HCH	2	84.6	5.2	95.2	3.9	99.0	2.4
Hexaconazole	2	101.3	7.1	106.3	4.0	101.6	3.8
Imidacloprid	5	92.9	3.9	98.1	18.3	95.1	6.4
Indoxacarb	5	74.9	6.7	110.3	10.9	93.3	3.2
Iprodione	5	93.2	9.4	102.1	6.5	107.5	3.6
Isazofos	2	97.4	97.7	103.6	3.2	101.7	6.6
Isocarbophos	2	106.2	7.8	102.3	1.8	103.8	7.9
Isofenphos-methyl	2	98.3	8.9	96.9	2.9	99.2	7.1
Isoprocarb	2	85.2	7.6	90.5	4.4	93.5	8.6
Isoxathion	2	90.8	8.1	99.1	4.1	91.3	2.1
Kresoxim-methyl	2	74.7	8.2	94.4	4.0	93.7	6.8
Malaoxon	2	97.6	9.6	96.2	5.5	92.5	3.0
Malathion	2	89.1	8.9	104.5	6.8	93.4	7.2
Mepanipyrim	2	98.6	7.8	100.1	2.7	91.3	4.7
Metalaxyl	2	104.3	9.5	93.7	4.3	98.1	3.4
Methacrifos	2	103.2	10.3	85.7	2.6	96.9	4.6
Methamidophos	2	80.1	7.6	77.8	5.9	80.6	7.2
Methomyl	5	76.9	6.4	109.7	8.4	96.4	4.4
Monocrotophos	2	86.7	6.8	101.0	2.5	95.7	4.3
Myclobutanil	2	85.4	5.2	88.6	9.9	82.0	4.0
Naled	2	97.8	3.3	99.2	6.8	92.2	1.3
Napropamide	10	91.4	8.2	102.6	1.2	93.8	6.1
Omethoate	2	78.3	7.5	88.0	6.5	86.4	5.1
Paclobutrazol	2	93.7	6.3	104.4	1.5	96.2	7.4
Parathion	2	110.8	6.4	104.9	2.5	97.3	5.8
Parathion-methyl	2	101.3	5.8	92.6	6.7	104.3	4.0
Penconazole	2	99.5	7.9	93.9	2.9	92.5	4.7
Pendimethalin	2	88.3	4.6	106.3	1.9	104.3	6.9
Phorate	2	105.6	8.5	98.9	2.6	93.3	5.5
Phorate sulfone	2	89.5	7.8	91.6	5.9	92.5	6.2
Phorate sulfoxide	2	90.3	6.7	94.8	2.9	101.6	8.4
Phosalone	2	91.8	9.1	117.7	1.7	94.3	4.9
Phosmet	5	93.2	7.5	108.9	9.0	97.8	6.3
Phoxim	5	91.5	3.5	102.2	10.1	88.7	6.1
Prochloraz	5	89.3	4.4	93.7	17.4	95.7	8.1
Procymidone	10	92.6	7.3	91.2	4.7	97.2	6.7

Profenofos	2	88.2	6.7	98.2	4.2	94.5	7.1
Propiconazole	2	93.7	5.1	103.6	2.6	101.2	3.8
Pyraclostrobin	5	90.5	2.4	113.3	6.9	91.5	4.0
Pyridaben	2	82.4	7.2	96.4	4.3	94.7	5.5
Pyrimethanil	2	103.6	4.8	103.7	7.1	99.3	4.8
Quinoxyfen	2	99.7	6.3	90.0	3.2	96.8	2.7
Spirodiclofen	5	91.5	3.0	97.0	8.8	103.1	4.4
Tebuconazole	2	94.5	6.5	99.7	6.2	88.3	6.6
Tecnazene	2	94.7	7.7	93.7	3.1	98.2	5.7
Terbufos	2	110.2	10.3	94.3	1.8	93.8	4.2
Terbufos sulfone	2	102.4	7.1	94.7	2.5	96.3	6.4
Tetramethrin	2	98.5	6.6	96.6	4.7	92.1	7.5
Thiamethoxam	5	95.4	3.0	92.1	10.8	90.3	3.7
Thiobencarb	2	89.4	7.9	87.6	3.7	106.1	5.7
Thiophanate-methyl	5	85.4	3.7	91.3	8.1	93.8	6.1
Triadimefon	2	95.7	6.5	95.6	1.5	89.6	4.3
Triazophos	2	96.3	8.2	102.5	2.9	98.4	3.2
Tridemorph	5	105.5	4.4	115.3	12.2	96.9	5.0
Uniconazole	5	90.5	2.9	86.8	20.6	92.3	6.3
Vamidothion	5	85.7	5.2	97.6	10.4	92.4	4.0
Vinclozolin	2	100.2	7.5	91.3	1.6	92.5	5.6
3-Hydroxycarbofuran	5	87.5	2.9	100.6	8.9	94.4	4.0
